# In-vitro antibacterial activity of methanolic extract of *Eucalyptus tereticornis* and *Ruta chalepensis* against selected pathogenic bacteria

**DOI:** 10.1016/j.vas.2026.100639

**Published:** 2026-03-29

**Authors:** Hailemariam Mihiretie Goshu, Sisay Alemu Mamo, Bruk Abraha Fitwi, Welelew Edmew Worku, Yeshiwas Wale Kasa, Yihenew Getahun Ambaw, Alubel Alemu Habesha, Simegnew Adugna Kallu, Ambachew Motbaynor Wubaye

**Affiliations:** a College of Veterinary Medicine, Haramaya University, P. Box 138, Dire Dawa, Ethiopia; b College of Agriculture and Environmental Science, Debre Tabor University, P. Box 272, Debre Tabor, Ethiopia

**Keywords:** Antibacterial activity, Ethiopia, *Eucalyptus tereticornis*, Minimum inhibitory concentration, Pathogenic bacteria, *Ruta chalepensis*

## Abstract

The rise of antimicrobial resistance is a critical global threat to both human and animal health, intensifying the search for novel antibacterial compounds from medicinal plants to supplement or replace failing conventional drugs. This study evaluated the *in-vitro* antibacterial activity of methanolic extracts from *Eucalyptus tereticornis* and *Ruta chalepensis* against selected pathogenic bacteria. Methanolic extracts were prepared from the bark and leaves of *E. tereticornis* and the aerial parts of *Ruta chalepensis*. The antibacterial potential was tested against *Escherichia coli* (ATCC 25,922), *Pseudomonas aeruginosa* (ATCC 27,853), and *Staphylococcus aureus* (ATCC 25,923) using the disc diffusion method, while Minimum Inhibitory Concentration (MIC) and Minimum Bactericidal Concentration (MBC) were determined via the broth dilution assay. Qualitative phytochemical screening was also conducted to identify secondary metabolites. The extraction yields were 35.4% (leaf) and 29 % (bark) for *E. tereticornis*, and 20 % for *R. chalepensis*. Among the extracts, *Ruta chalepensis* exhibited the strongest antibacterial activity, particularly against *S. aureus*, with a mean zone of inhibition of 18.7 ± 0.7 mm and a notably low MIC of 1.95 mg/ml. Although all extracts demonstrated dose-dependent activity, Gram-positive *S. aureus* was found to be more susceptible than the Gram-negative strains. These findings demonstrate that *R. chalepensis* and *E. tereticornis* possess significant antibacterial properties against standard pathogenic strains, providing a scientific basis for their traditional use and highlighting their potential as sources for new antimicrobial agents.

## Highlights

• Antibacterial activity of *E. tereticornis* and *R. chalepensis* was evaluated.
• *Ruta chalepensis* showed the highest potency against *S. aureus* (18.7 ± 0.7 mm).
• All extracts contained alkaloids, flavonoids, tannins, and saponins.
• Gram-positive *S. aureus* was more susceptible than Gram-negative strains.
• These plants represent potential sources for future antimicrobial agents.

## 1 Introduction

Nature has been a primary source of medicinal agents for thousands of years and since the dawn of mankind (Jain et al., 2010), and natural sources have been used as a foundation for numerous modern drugs (Aylate et al., 2017). The most dominant natural medicine source is plants, due to their chemical and structural variety and the biodiversity of their components (Mathur & Hoskins, 2017); many of these isolations were based on the uses of the agents in traditional medicine (Rahman et al., 2023). Plant-based ethno-veterinary medicine is also extensively used across the world since livestock raising is an integral part of most people's livelihoods (Rahman et al., 2023). To keep animals healthy, traditional healing practices have been applied for centuries and have been passed down orally from generation to generation. Before the introduction of western medicine, all livestock keepers relied on these traditional practices. At least 80 % of people in developing countries depend largely on these practices to control and treat various diseases that affect both animals and humans (Dinbisoet al., 2022).

Plants contain phytochemicals in response to environmental factors such as attack by herbivores, abiotic stress, or interspecific interactions (Álvarez-Martínez et al., 2021). Phytochemicals are naturally occurring substances and provide health benefits. Alkaloids, flavonoids, tannins, phenols, saponins, steroids, glycosides, and terpenes are some of the plants' major secondary metabolites that have antioxidant, anti-inflammatory, anti-cancer, and anti-microbial properties (Marami et al., 2021). Numerous effective and powerful medicines can be obtained from plants. This is due to the presence of different bioactive compounds in different plant parts, and each of these compounds possesses different therapeutic activity (Yirdaw & Kassa, 2023).

Plant medications in Ethiopia are still the most important and sometimes the only sources of treatments for approximately 80 % of the human and >90 % of the animal population (Kalayou et al., 2012). Since Ethiopia is located in the Horn of Africa, it is rich in flora, so it developed medicinal uses for many of the plant species. The flora has greater than 6500 vascular plant species, of which about 12 % are endemic (Yirgu et al., 2019). Ethiopia also has the largest livestock population in Africa and it plays very important roles as sources of draft power, nutrition, cash income, employment, and poverty alleviation (Jain et al., 2010). Even though Ethiopia has the largest livestock population, it has one of the world's lowest unit outputs. Animal diseases are considered as a major health problem and cause a significant economic loss in the country (Albe et al., 2016). However, enormous efforts to control infectious diseases caused by bacteria, fungi, viruses, and parasites continue to pose a challenging threat to livestock health. This is due to a shortage of new medicines and the development of antimicrobial resistance (Kalayou et al., 2012) and the rise of extensively resistant strains (Yirdaw & Kassa, 2023).

Pathogenic bacteria refers to microorganisms that have developed resistance to at least one antibiotic in three or more different antimicrobial classes (Exner et al., 2017). This resistance is considered a modern microbial trait that results from unsuccessful or prolonged exposure to antibiotic treatments (Palma et al., 2020). It is one of the most significant threats to human and animal health globally (Palma et al., 2020), disseminating rapidly and complicating the treatment of bacterial infections in both humans and animals. Even advanced medications have proven ineffective against these resistant bacteria, as they continue to develop resistant mechanisms (Marami et al., 2021).

Bacteria such as *Staphylococcus aureus* and *Escherichia coli* naturally exhibit resistance to many antibiotic agents (Dinbiso et al., 2022), while *Pseudomonas aeruginosa* can develop resistance by acquiring chromosomal mutations and resistance (AR) genes (Botelho et al., 2019). These microorganisms are not only capable of resistance but are also known to be extensively drug-resistant and pan-drug-resistant bacteria (Romha et al., 2018). The renewed interest in medicinal plants with antimicrobial properties has emerged in response to ongoing issues related to antibiotic use and the rising prevalence of resistant strains among various pathogenic bacteria (Doughari et al., 2009). A key area of focus for medicinal plant extracts is their potential to inhibit the growth and decrease the populations of these more dangerous pathogens (Kang et al., 2011).

*Eucalyptus tereticornis* (*E. tereticornis*) is the most common eucalyptus genus of the Myrtaceae family. The common name of *E. tereticornis* is forest red gum; it grows to a height of about 20 to 50 m, and trunk girth is about 2 m. It also possesses strong medicinal properties like other species of the family (Kiran et al., 2020), which is used traditionally for the treatment of wounds, boils, and other ailments. *Eucalyptus* species are known to produce numerous volatile compounds in large amounts (Badrunnisa et al., 2012), used in arthritis, asthma, burns, fever, influenza, sore throat, malaria, wounds, and pharyngitis treatments (Kiran et al., 2020).

*Ruta chalepensis* (*R. chalepensis*) is primarily shrubby plants that are native to the Mediterranean region and usually grow on rocky slopes (Ouerghemmi et al., 2017). Extract of *R. chalepensis* is used as a medicinal agent against the evil eye and for spiritual cleansings, whereas the infusions of its fresh leaves are widely used as a treatment for analgesic activity, gastric disorders, headache, and rheumatism. It is also used as a diuretic, treatment of inflammation, ulcer, hypotension, reproductive problems like menstrual disorders, and anti-spasmodic activities. This plant has also been used as a mosquito repellent, an antidote to snake and scorpion venom poisoning, and as a poultice for bites and stings (Doughari et al., 2009; Muthuramu et al., 2020).

**Fig. 1:**
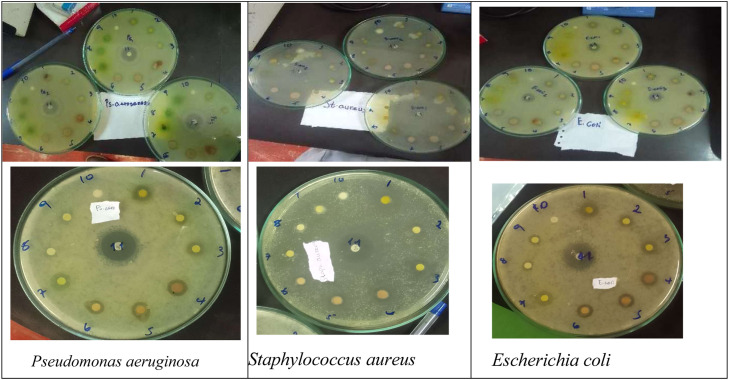
**Representative agar disc diffusion plates demonstrating the antibacterial activity of methanolic plant extracts.** The clear zones around the filter paper discs indicate the zones of inhibition (ZOI) against the tested pathogens. **(Top)** Overview of triplicate testing used to ensure the reliability and replicability of the experimental data. **(Bottom)** Close-up view of individual plates showing the susceptibility of *Pseudomonas aeruginosa* (ATCC 27,853), *Staphylococcus aureus* (ATCC 25,923), and *Escherichia coli* (ATCC 25,922), to varying concentrations of *Ruta chalepensis* and *Eucalyptus tereticornis* extracts.

Despite their known uses, there is a lack of comparative data on the efficacy of methanolic extracts from these species specifically sourced from the Harar region, Ethiopia. Therefore, the aim of this study was to evaluate the in-vitro antibacterial activity of methanolic extracts of *E. tereticornis* (bark and leaf) and *R. chalepensis* (aerial) collected from Harar, Ethiopia and tested against three significant pathogenic bacteria: *E. coli* (ATCC 25,922), *Pseudomonas aeruginosa* (ATCC 27,853), and *Staphylococcus aureus* (ATCC 25,923) (Fig. 1).

## 2 Materials and methods

### 2.1 Description of the study area

The plant material extraction and *in-vitro* antibacterial evaluations were carried out at the Haramaya University College of Agriculture and Environmental Science laboratory. Haramaya is located in the Haramaya district, approximately 527 km east of Addis Ababa (Serda & Abdi, 2018). The area’s elevation ranges from 1800 to 2345 m above sea level, with an average altitude of 2047 m, situated at 9°26′N latitude and 42°3′E longitude. The district receives an average annual rainfall of approximately 900 mm and is characterized by two main ecological zones: midland (66.5 %) and lowland (33.5 %).

### 2.2 Study design

An *in-vitro* experimental study was conducted from November 2023 to March 2024 to evaluate the antibacterial activity of methanolic extracts of the bark and leaves of *E. tereticornis* and the aerial parts of *R. chalepensis* against standard strains of *S. aureus* (ATCC 25,923), *E. coli* (ATCC 25,922), and *P. aeruginosa* (ATCC 27,853).

### 2.3 Collection and extraction of plant materials

The plant species used in this study, *E. tereticornis* (bark and leaf) and *R. chalepensis* (aerial parts), were selected based on previous reports of their antibacterial activity (Kiran et al., 2020; Bekkar et al., 2022). Fresh leaves and bark of *E. tereticornis* and aerial parts of *R. chalepensis* were collected from Hakim Woreda in Harar City, Ethiopia. The species were identified and authenticated at the Department of Plant Sciences, College of Agriculture, Haramaya University, and assigned voucher numbers HAILE/8/23 and HAILE/9/23, respectively. Latex gloves were worn during collection to minimize contamination risks. The samples were spread on clean sheets in shaded areas at room temperature to air-dry for two weeks. Once dried, the plant parts were ground into a fine powder using an electric grinder, sieved, and weighed for extraction.

### 2.4 Crude extract preparation

Crude extracts were prepared according to the procedure described by Marami et al. (2021). For each sample, 100 g of powder were placed in a flat-bottom flask with 500 mL of 99.9 % methanol and macerated for 24 h on a rotary shaker at 80 rpm. The extracts were filtered through Whatman No 1 filter paper and concentrated using a vacuum rotary evaporator at 50 °C to remove the solvent.

The percentage yield for each extract was determined according to the method by Dinbisoet al. (2022) using the following formula.

(100) *Percentage* extract yield (%) = Weight of extract/ Weight of dried powder ×

The final concentrates were stored in secure, labeled bottles at 4 °C until testing.

### 2.5 Preliminary bioactive metabolite screening

The presence of secondary metabolites namely; saponins, tannins, alkaloids, steroids, cardiac glycosides, phenolic compound, flavonoids, and triterpenes were assessed by using Qualitative bioactive screening tests.^8^ Briefly; the presence of Saponins was assessed by using foam test; accordingly, 0.5 mg of the extract was shaken with 2 mL of distilled water then if foam is produced and persisted for at least 10 min, it confirms the presence of Saponins. While the presence Tannins was tested by Ferric Chloride test; hence, 0.25 g of extract was heated separately in a water bath with 10 mL of distilled water. After filtration, three drops of 0.1 % ferric chloride solution were added to the filtrate. The appearance of a blue, dark blue, green, or blue-green coloration or precipitates indicated the presence of tannins. Steroids were tested by adding 2 mL of acetic anhydride to 0.5 g of the plant extract in a test tube, followed by 2 mL of sulfuric acid. Violet to blue or green color change signified the presence of steroids. Alkaline Reagent Test method was used to test the presence of Flavonoids; the extracts were treated with drops of sodium hydroxide solution. The formation of a deep yellow color, which turns colorless upon adding dilute acid, indicated flavonoids. Phenolic compounds were tested through phenol test by adding 2 % iron chloride (FeCl₃) to 0.5 g of crude extract. The development of a bluish-green or black coloration suggested the presence of phenolic compounds. Keller-Kiliani test were used to Cardiac Glycosides, hence; 1.25 mg of the plant extracts were combined with 0.5 mL of chloroform and thoroughly mixed with 0.5 ml of concentrated H2SO4. The reddish-brown color at the interface indicates the presence of a steroidal ring, the glycone part of glycoside. Dragendorff test were used to test alkaloids; hence, 200 mg of plant extracts were boiled in 10 mL of methanol and filtered, the after 1 % HCl and six drops of Dragendorff reagent were added, formation of a brownish-red precipitate confirmed the presence of alkaloids. The Liebermann–Burchard’s testwere used to test Triterpenoids; accordingly, 2 mg of dried extract was dissolved in acetic anhydride and heated to boiling. After cooling, 1 mL of concentrated sulfuric acid was added carefully along the side of the test tube. The appearance of a violet-colored ring indicated the presence of triterpenoids.

### 2.6 Test organisms

Standard strains of *S. aureus* (ATCC 25,923), *E. coli* (ATCC 25,922), and *P. aeruginosa* (ATCC 27,853) were obtained from the Haramaya University College of Veterinary Medicine Microbiology Laboratory. The strains were cultured on nutrient agar (Olajuyigbe et al., 2018; Gupta et al., 2014).

### 2.7 In-vitro antimicrobial activity

Antibacterial testing was performed using the disc diffusion method (Dinbiso et al., 2022). Mueller-Hinton agar (MHA) was sterilized at 121 °C for 15 min and poured into sterile petri dishes. Extracts were prepared in 5 % dimethyl sulfoxide (DMSO) at concentrations of 1000, 500, and 250 mg/mL. Sterile 6 mm filter paper discs were loaded with 20 μL of each extract and placed on MHA plates previously inoculated with the test organisms.

Bacterial turbidity was adjusted to the 0.5 McFarland standard (∼1.5 × 10⁸ CFU/mL) in sterile saline. The suspensions were spread evenly on MHA plates using sterile swabs. Following the application of extract discs and positive control discs (Ciprofloxacin, 5 µg), plates were incubated at 37 °C for 24 h. The diameters of inhibition zones were measured in millimeters using a caliper. All procedures were performed in triplicate.

### 2.8 Minimum inhibitory concentration (MIC) and minimum bactericidal concentration (MBC)

MIC and MBC values were determined using the tube dilution method (Bekkar et al., 2022). Serial two-fold dilutions were prepared in nutrient broth, yielding concentrations from 500 mg/mL down to 1.95 mg/mL. Each tube was inoculated with 100 µL of bacterial suspension (1.5 × 10⁶ CFU/mL). Positive (broth + bacteria) and negative (broth + extract) controls were included. After 24 h of incubation at 37 °C, the lowest concentration with no visible turbidity was recorded as the MIC. To determine the MBC, samples from tubes showing no growth were sub-cultured onto agar plates; the lowest concentration yielding no growth after 24 h was recorded as the MBC.

### 2.9 Data management and analysis

Data were recorded in Excel and analyzed using Stata Version 17. Results are presented as Mean ± Standard Error (Mean ± SE). Differences between means were assessed using one-way ANOVA, with significance set at *p* < 0.05.

## 3 Result

### 3.1 The percentage yields of the plant extracts

Among the macerates prepared from *E. tereticornis* and *R. chalepensis,* the leaf of *E. tereticornis* produced the highest yield at 35.4 %. In contrast, the bark of *E. tereticornis* and the aerial parts of *R. chalepensis* yielded 29 % and 20 %, respectively (Table 1). The crude extract from leaf and bark of *E. tereticornis* and aerial parts of *R. chalepensis* had deep green, coffee and dark green colour respectively .

**Table 1:** Extraction yield of leaf and bark *of E. tereticornis and, R. chalepensis*.

Plant species	Part used	Sample (g)	Yield (g)	Yield (%)	Extract colour	Extract consistency
*E. tereticornis*	Bark	100	29	29	Coffee colored	Semisolid
	Leaf	100	35.4	35.4	Deep green	Semisolid
*R. chalepensis*	Aerial	100	20	20	Dark green	Semisolid

Qualitative phytochemical analysis was performed on the plant extracts to detect various secondary metabolites. R. chalepensis tested positive for all tested secondary metabolites. Both the bark and leaf of E. tereticornis showed the presence of alkaloids, tannins, saponins, steroids, flavonoids, and cardiac glycosides. Additionally, the leaf extract of E. tereticornis was positive for phenolic compounds, while the bark extract contained triterpenes (Table 2)

**Table 2:** Secondary metabolites present in the extraction of leaf and bark of *E. tereticornis and, R. chalepensis*.

Phytochemical	*Eucalyptus tereticornis*		*Ruta chalepensis(arial)*
	Bark	Leaf	
Saponins	+	+	+
Tannins	+	+	+
Steroids	+	+	+
Flavonoids	+	+	+
Cardiac glycosides	+	+	+
Alkaloids	+	+	+
Triterpens	+	-	+
Phenolic compound	-	+	+

### 3.2 Antibacterial activities of crude methanol extracts of *E. tereticornis* (leaf and bark) and *R. chalepensis* (aerial part)

The inhibition zone result (mm, diameter) created by antibacterial activities of crude methanol extracts of *E. tereticornis* (leaf and bark) and *R. chalepensis* (aerial part) were measured. All plant extracts at all tested concentrations showed good antibacterial activity against tested bacteria (Fig. 1). *R. chalepensis* at 100 % (18.3 ± 0.9 mm) and 50 % (16.7 ± 0.3 mm) shows comparable antibacterial activity with standard antibiotic (Ciprofloxacin 5 µg per disc) (18.3 ± 0.7 mm) against *E. coli* (ATCC25922). The highest zone of inhibition 18.7 ± 0.7 mm was recorded from *R. chalepensis* against *S. aureus* (ATCC 25,923). The lowest antibacterial activity was observed in 25 % bark of *E. tereticornis* against *E. coli* (ATCC25922) (13.0 ± 0.0 mm) (Table 3).

**Table 3:** Mean ±SE zone of inhibition of extracts against multidrug resistant bacteria at various concentrations.

Plants	Concentrations (mg/ml)	*S. aureus* (ATCC25923)	*E. coli* (ATCC25922)	*P.aeruginosa* (ATCC27853)
*E.* *tereticornis* (bark)	1000	14.0 ± 0.6^l^	15.0 ± 0.6^a^	14.7 ± 0.3^fg^
	500	15.7 ± 0.3^n^	14.0 ± 0.6	16.7 ± 0.3^i^
	250	14.3 ± 0.3^pq^	13.0 ± 0.0	15.7 ± 0.3^k^
*E.* *tereticornis (leaf)*	1000	16.3 ± 0.3^lm^	15.7 ± 0.3^a^	14.3 ± 0.3^fh^
	500	16.3 ± 0.3^no^	16.7 ± 0.3^c^	13.7 ± 0.9^j^
	250	14.0 ± 0.0^pr^	15.0 ± 0.0^d^	13.3 ± 0.3
*R.* *chalepensis* (aerial)	1000	17.3 ± 0.3^m^	18.3 ± 0.9^b^	15.7 ± 0.3^gh^
	500	18.7 ± 0.7°	16.7 ± 0.3^c^	14.7 ± 0.3^ij^
	250	15.0 ± 0.6^qr^	16.0 ± 0.0^d^	14.3 ± 0.3^k^
Ciprofloxaci n	5µg	26.0 ± 1.0	18.3 ± 0.7^bc————–^	23.7 ± 0.3
DMSO	5 %	0.0 ± 0.0	0.0 ± 0.0	0.0 ± 0.0

The values are Mean ± *S*.E.M (*n* = 3); significant at *P* < 0.05; comparison between plants of the.

Same concentration; column values with similar letters indicates the values are not significant (has similar value but may not necessarily equal).

### 3.3 Minimum inhibition and bactericidal concentration of the plant extracts

All the plant extracts had shown inhibitory and bactericidal activity and their minimum inhibitory concentration ranges from 1.95 mg/ml to 7.8 mg/ml while their minimum bactericidal concentration were between 3.90 mg/ml and 15.6 mg/ml*. R. chalepensis* (aerial part) showed strong inhibitory and bactericidal activity with 1.95 mg/ml minimum inhibitory concentration and 3.90 mg/ml minimum bactericidal concentration against *S. aureus* (ATCC 25,923) while *E. tereticornis* (leaf) and *R. chalepensis* (aerial) showed relatively weak inhibitory and bactericidal activity with 7.8 mg/ml minimum inhibitory concentration and 15.6 mg/ml minimum bactericidal concentration against *P. aeruginosa* (ATCC27853) (Table 4).

**Table 4:** Minimum inhibition concentration and minimum bactericidal concentration (mg/ml) of leaf and bark of *E. tereticornis and, R. chalepensis* extracts against the tested organism.

Bacteria	Plants used	MIC (mg/ml)	MBC (mg/ml)
*E.coli* (ATCC25922)	*E. tereticornis*(bark)	3.9	7.8
	*E. tereticornis* (leaf)	3.9	7.8
	*R. chalepensis* (aerial part)	3.9	7.8
*P.aeruginosa* (ATCC27853)	*E. tereticornis* (bark)	3.9	7.8
	*E. tereticornis* (leaf)	7.8	15.6
	*R. chalepensis* (aerial part)	7.8	15.6
*S.aureus* (ATCC 25,923)	*E. tereticornis* (bark)	3.9	7.8
	*E. tereticornis* (leaf)	3.9	7.8
	*R. chalepensis* (aerial part)	1.95	3.90

* MIC: minimum inhibitory concentration; MBC: minimum bactericidal concentration.

## 4 Discussion

The development of new antimicrobial agents from plant sources is a vital area of research due to the persistent rise of antibiotic resistance. In the present study, the methanolic extracts of *E. tereticornis* and *R. chalepensis* demonstrated varying degrees of antibacterial activity against standard strains of *S. aureus, E. coli*, and *P. aeruginosa.*

The final crude extract yield obtained from the bark and leaf of *E. tereticornis* was 29 % and 35.4 %, respectively. Although there is no previous report specifically detailing the percentage yield from the bark of *E. tereticornis*, the yield obtained from the leaves in this study is highly comparable with the 37.05 % yield from methanol extraction reported by Badrunnisa (Badrunnisa et al., 2012). However, this finding is significantly higher than the 10 % yield of n-hexane extraction reported by Manikkoth et al. (Manikkoth et al., 2017). This variation in extraction yield is likely attributable to the difference in the polarity of the solvents used; in this study, methanol was selected for its high polarity compared to n-hexane, and it is well-established that as solvent polarity increases, the efficiency of extracting polar plant constituents and the overall yield also increase (Murugan & Parimelazhagan, 2014; Nawaz et al., 2020). Furthermore, the crude extraction yield of *R. chalepensis* was 20 %, a result that aligns with the 19.39 % yield reported by Elizondo-Luévano et al. (Elizondo-Luévano et al., 2023), yet remains higher than the 1.25 % leaf yield reported by Fakhfakh et al. (Ghaffar et al., 2015). Such discrepancies might be due to variations in the plant parts utilized or the geographical source of the samples.

The qualitative phytochemical screening of *E. tereticornis* (both bark and leaf) revealed the presence of alkaloids, tannins, saponins, steroids, flavonoids, and cardiac glycosides. Specifically, the bark was positive for triterpenes and negative for phenolic compounds, while the leaf was positive for phenolic compounds and negative for triterpenes. These results are in agreement with the findings of Trivedi and Maurya et al. (Maurya et al., 2016; Trivedi, 2021), who identified a similar profile of secondary metabolites in this species. Similarly, *R. chalepensis* (aerial part) tested positive for all secondary metabolites evaluated, consistent with the reports of Marami et al. (Marami et al., 2021) and Workie and Desta (Workie & Desta, 2022), who confirmed the presence of alkaloids, flavonoids, phenolic compounds, saponins, and tannins.

The antibacterial efficacy observed in this study can be scientifically linked to the presence of these specific metabolites. For instance, flavonoids are reported to inhibit bacterial growth by forming complex bonds with both soluble proteins and the bacterial cell wall; more specifically, they have been shown to inhibit DNA gyrase and disrupt the microbial electron transport chain, thereby interfering with energy metabolism (Cushnie & Lamb, 2005). In a similar fashion, tannins exert their antimicrobial influence by inactivating essential microbial enzymes, such as those involved in oxidative phosphorylation, and by sequestering the iron necessary for bacterial survival (Scalbert, 1991). Furthermore, tannins are capable of inducing the precipitation of membrane proteins, which triggers the leakage of intracellular constituents and eventually leads to cell lysis (Shimada, 2006). This diversity of secondary metabolites provides a robust theoretical framework for the extracts' potency. Even in the absence of quantitative GC–MS profiling, the observed zones of inhibition in our results strongly align with the documented bioactivities of these chemical classes.

The methanolic extract from the bark of *E. tereticornis* exhibited significant antibacterial activity against all tested pathogens, particularly against *P. aeruginosa* (ATCC 27,853) with a 16.7 ± 0.3 mm zone of inhibition and *S. aureus* (ATCC 25,923) with a 15.7 ± 0.3 mm zone. While literature regarding the activity of the bark specifically against *P. aeruginosa* (ATCC 27,853) is scarce, the activity against *S. aureus* observed here aligns with the 17 mm reported by Jain et al. (Jain et al., 2010). Conversely, the least susceptible strain was *E. coli* (ATCC 25,922) with a 15.0 ± 0.6 mm zone, which is lower than the 26 mm reported by Jain et al. (Jain et al., 2010). This variation in results compared to previous studies may also stem from differences in the plant’s age and the season of collection; our samples were harvested from older trees during the rainy season, a period during which the concentration of essential secondary metabolites is often diluted or reduced due to physiological and environmental factors (Farias et al., 2023).

The observed resistance patterns in both *E. coli* and *P. aeruginosa* are largely governed by their Gram-negative cell wall architecture. Both species possess an asymmetrical outer membrane composed of lipopolysaccharides (LPS) that serves as a robust permeability barrier against many antibacterial agents (Nikaido, 1994). However, the higher susceptibility of *P. aeruginosa* compared to *E. coli* in this study suggests that the bioactive compounds in *E. tereticornis* bark may more effectively bypass the specific porin channels of *P. aeruginosa* or better counteract its restricted membrane permeability (Breidenstein et al., 2011). Furthermore, while both organisms utilize efflux pump systems to expel toxins, the varying efficacy suggests a higher affinity of the extract's phytochemicals for the molecular targets within *P. aeruginosa* compared to *E. coli* (Delcour, 2009).

The antibacterial activity of the leaf extract of *E. tereticornis* against *P. aeruginosa* (ATCC 27,853) was recorded at 14.3 ± 0.3 mm. This finding is in close alignment with the zones of 14.5 ± 0.3 mm and 15.25±0.00 mm reported by Siddique et al. (Siddique et al., 2020) and Badrunnisa and Pai (Badrunissa & Pai, 2023), respectively. Furthermore, the zone of inhibition produced by the leaf extract against *E. coli* (ATCC 25,922) was 16.7 ± 0.3 mm. This result remains comparable with the 18 mm reported by Jain et al. (Jain et al., 2010), although it is notably lower than the 24.49 mm reported by Fawad et al. (Fawad et al., 2012) and higher than the 12 mm observed by Bachheti et al. (Bachheti et al., 2011). Such variations in inhibitory zones across different studies are frequently attributed to the chemical plasticity of the genus *Eucalyptus*, where the concentration of active constituents can fluctuate based on the specific extraction methodology and the geographical origin of the plant material.

Generally, the leaves of *E. tereticornis* demonstrated a significantly more potent antibacterial effect than the bark. This superior efficacy is likely due to the distinct physiological roles and chemical profiles of these two plant parts. Leaves serve as the primary site for photosynthesis and gas exchange, metabolic processes that actively stimulate the biosynthesis of essential oils, flavonoids, and phenolic compounds as a proactive defense mechanism against microbial pathogens and environmental stressors (Yirdaw & Kassa, 2023).

From a mechanistic perspective, the higher concentration of volatile terpenes—such as 1,8-cineole—found in the leaves may facilitate better penetration of the bacterial cell envelope. These lipophilic compounds are known to disrupt the structural integrity of the cell membrane, leading to the loss of electrolytes and vital intracellular proteins (Mulyaningsih et al., 2010). Furthermore, while the bark serves a protective, structural function and contains significant levels of tannins, the leaves often possess a more diverse "phytochemical cocktail" that allows for synergistic interactions between different classes of metabolites, thereby enhancing their overall bactericidal capacity compared to the more fibrous bark tissue.

The methanolic extract of *R. chalepensis* exhibited substantial antibacterial potency against *S. aureus* (ATCC 25,923), *P. aeruginosa*, and *E. coli*, with recorded zones of inhibition of 18.7 ± 0.7 mm, 15.7 ± 0.3 mm, and 18.3 ± 0.9 mm, respectively. These findings are highly comparable with the results reported by Priya et al. (Priya et al., 2009), who observed zones of 14 mm, 11 mm, and 16 mm for the same bacterial species. Conversely, our results contrast sharply with the study by Babu-Kasimala et al. (Babu-Kasimala et al., 2014), which reported significantly smaller inhibitory zones of 7.5 mm and 8.0 mm for acetone extracts of *R. chalepensis* against *E. coli* and *S. aureus*.

This marked variation in antibacterial efficacy is likely attributable to the choice of extraction solvent. In this study, methanol was utilized, which possesses a higher dielectric constant and polarity compared to acetone. Consequently, methanol is more effective at exhausting the plant material of a broader spectrum of bioactive polar compounds, leading to a more potent extract (Vaghasiya & Chanda, 2007). This underscores the critical role of solvent selection in the recovery of secondary metabolites such as quinoline alkaloids and coumarins, which are prevalent in the *Ruta* genus and responsible for membrane disruption.

Generally, when compared with both the bark and leaf extracts of *E. tereticornis, R. chalepensis* demonstrated a significantly superior antibacterial profile across all tested concentrations. This enhanced bioactivity may be explained by the superior phytochemical diversity and bioavailability inherent in the aerial portions (stems, leaves, and flowers) of *R. chalepensis*. It is also likely the result of "phytochemical synergism," where the simultaneous presence of multiple compound classes—such as alkaloids, flavonoids, and phenols—targets different metabolic pathways in the bacteria at once. For instance, while one compound may increase cell wall permeability, another may simultaneously inhibit intracellular enzymes or DNA replication (Gilbert & Alves, 2003). Furthermore, the aerial parts of *R. chalepensis* provide a more complex chemical matrix that may enhance the solubility and absorption of active agents across the bacterial cell envelope compared to the more specialized tissue of *E. tereticornis* bark or leaves.

The Minimum Inhibitory Concentration (MIC) and Minimum Bactericidal Concentration (MBC) for both the bark and leaf extracts of *E. tereticornis* were determined to be 3.9 mg/mL and 7.8 mg/mL for *E. coli* and *S. aureus*, respectively. Notably, these values doubled in the case of *P. aeruginosa*. This reduced sensitivity observed in *P. aeruginosa* is likely attributable to its formidable intrinsic resistance mechanisms, most notably its capacity for biofilm formation. These multicellular communities produce an extracellular polymeric substance (EPS) matrix that acts as a physical and chemical shield, significantly impeding the penetration of plant-derived antibacterial agents (Haidar et al., 2024).

For *R. chalepensis*, the MIC/MBC values were recorded at 3.9/7.8 mg/mL for *E. coli*, 7.8/15.6 mg/mL for *P. aeruginosa*, and a highly potent 1.95/3.90 mg/mL for *S. aureus*. While these values demonstrate significant activity, they are slightly higher than those reported by previous investigators (Al-Majmaie et al., 2021). This discrepancy is likely a consequence of the specific agro-climatic conditions characterizing the collection site in Harar. It is well-documented in ethnobotanical literature that plants subjected to the physiological stress of higher altitudes and lower temperatures often exhibit an up-regulation in the synthesis of secondary metabolites. Such environmental stressors stimulate the production of more concentrated bioactive compounds as an adaptive survival strategy, which in turn enhances the antibacterial potency of the resulting extracts (Sukmawaty et al., 2024).

Furthermore, the relationship between the MIC and MBC values in this study is scientifically significant. In most instances, the MBC was only two fold higher than the MIC, suggesting that the extracts possess a bactericidal rather than a merely bacteriostatic mode of action. A ratio of MBC/MIC ≤4 is generally accepted as an indicator of bactericidal activity, which further substantiates the potential of these plants as sources of definitive antimicrobial agents (French, 2006).

Crucially, the MIC of *R. chalepensis* against *S. aureus* (1.95 mg/mL) demonstrates an exceptional inhibitory potential. While this study utilized standard ATCC reference strains to establish a controlled, replicable, and rigorous baseline for evaluation—thereby addressing the requirement for standardized methodology—the significant efficacy observed strongly positions these extracts as prime candidates for subsequent testing against clinical multidrug-resistant (MDR) phenotypes. Indeed, the proximity of these inhibitory values to established pharmacological benchmarks, such as the MIC of vancomycin against specific MDR strains (Scalbert, 1991), underscores the therapeutic promise of these traditional medicinal plants. These findings contribute valuable data to the urgent global search for novel antibiotic scaffolds derived from natural products."

### 4.1 Limitations of the study

Despite the promising findings, certain limitations of this study must be addressed. First, while qualitative phytochemical screening confirmed the presence of key bioactive groups like tannins and flavonoids, the study lacked quantitative characterization via GC–MS or HPLC. This was primarily due to resource constraints and limited access to high-resolution analytical instrumentation at the time of the study. However, the qualitative results provided a necessary preliminary profile that aligns with the observed antibacterial zones.

Secondly, the investigation utilized standard ATCC reference strains rather than clinical multidrug-resistant (MDR) isolates. While this was criticized by the reviewers, the use of standardized strains was a deliberate choice to ensure the replicability and reliability of the baseline data. By using quality-controlled strains, we established a rigorous foundation of the extracts' potency before moving to more variable clinical isolates in future work. Lastly, the study did not include electron microscopy (SEM/TEM) to visualize the morphological changes in the bacteria. While such imaging would provide visual evidence of cell wall lysis, the consistent MBC/MIC ratios obtained through broth dilution provide strong indirect evidence of the bactericidal nature of the extracts. These limitations do not invalidate the current findings but rather define the scope for future, more advanced pharmacological trials.

## 5 Conclusions

In conclusion, this study demonstrates that the methanolic extracts of *Eucalyptus tereticornis* and *Ruta chalepensis* possess significant *in-vitro* antibacterial activity against common pathogenic bacteria, including *S. aureus, E. coli*, and *P. aeruginosa*. The results indicate that *R. chalepensis* generally exhibits a more potent inhibitory effect compared to *E. tereticornis*, likely due to a higher diversity of synergistic phytochemicals. The low MIC and MBC values, particularly against *S. aureus*, underscore the potential of these plants as viable sources for the development of natural antimicrobial agents.

While the use of standard reference strains provided a rigorous baseline for this initial evaluation, future studies should focus on testing these extracts against clinical multidrug-resistant isolates and performing detailed chemical profiling via GC–MS to identify the lead bioactive molecules. Overall, these findings provide a scientific rationale for the traditional use of *E. tereticornis* and *R. chalepensis* in treating infectious diseases and highlight their importance in the ongoing search for novel antibiotic scaffolds to combat global antimicrobial resistance.

## Funding

This research did not receive any specific grant from funding agencies in the public, commercial, or not-for-profit sectors.

### Ethical statement

This study was conducted *in-vitro* using botanical samples and standard bacterial reference strains. No human participants or live animals were involved in this research; therefore, formal ethical approval was not required for this study.

As this was an *in-vitro* study using botanical samples and standard bacterial reference strains, no human participants or live animals were involved. Consequently, formal institutional ethical approval was not required. This has been explicitly stated in the revised manuscript.

## CRediT authorship contribution statement

**Hailemariam Mihiretie Goshu:** Writing – original draft, Data curation, Conceptualization. **Sisay Alemu Mamo:** Methodology, Data curation. **Bruk Abraha Fitwi:** Writing – review & editing. **Welelew Edmew Worku:** Writing – review & editing. **Yeshiwas Wale Kasa:** Data curation, Conceptualization. **Yihenew Getahun Ambaw:** Formal analysis, Data curation. **Alubel Alemu Habesha:** Writing – review & editing. **Simegnew Adugna Kallu:** Methodology, Conceptualization. **Ambachew Motbaynor Wubaye:** Supervision, Methodology, Formal analysis, Conceptualization.

## Declaration of competing interest

The authors declare that there is no competing interest.

## Data Availability

All the raw data generated, analyzed, and presented in this article are available from the corresponding author upon reasonable request.
